# Utilizing Red Spotted Apollo Butterfly Transcriptome to Identify Antimicrobial Peptide Candidates against *Porphyromonas gingivalis*

**DOI:** 10.3390/insects12050466

**Published:** 2021-05-18

**Authors:** Kang-Woon Lee, Jae-Goo Kim, Karpagam Veerappan, Hoyong Chung, Sathishkumar Natarajan, Ki-Young Kim, Junhyung Park

**Affiliations:** 1 Holoce Ecosystem Conservation Research Institute (HECRI), Hweongsung 25257, Gangwon-do, Korea; holoce@hecri.re.kr; 2 Graduate School of Biotechnology, Kyung Hee University, Yongin-si 17104, Gyeonggi-do, Korea; zxcv913@naver.com; 3 3BIGS Co. Ltd., 156, Gwanggyo-ro, Yeongtong-gu, Suwon-si 16506, Gyeonggi-do, Korea; karpagam@3bigs.com (K.V.); hychung@3bigs.com (H.C.); sathish@3bigs.com (S.N.); 4 Department of Genetics and Biotechnology, College of Life Science, Kyung Hee University, Yongin-si 17104, Gyeonggi-do, Korea

**Keywords:** *Parnassius bremeri*, transcriptome, antimicrobial peptide, *Porphyromonas gingivalis*, endangered species

## Abstract

**Simple Summary:**

Infections caused by bacteria, fungi, or viruses possess serious threat to human health and life. This is well realized in the current COVID-19 pandemic scenario. Antimicrobial peptides (AMPs) are a natural line of defense in many organisms, especially insects which survive in extreme niches. Here we identified AMPs from red spotted apollo butterflies found at high altitudes in Russia, China, and Korea. The larval development stage occurs on the months of December to April, when there are very low temperatures. The insects natural defense mechanism might contribute to withstand this condition, which is our point of interest, and we utilized the genomic information to identify AMPs from red spotted butterflies. The obtained AMPs were tested against a list of pathogenic bacteria and fungi. Finally, we obtained one promising candidate active against *Porphyromonas gingivalis,* a causative organism for periodontitis. With further validations, this could be a lead antimicrobial agent in future.

**Abstract:**

Classical antibiotics are the foremost treatment strategy against microbial infections. Overuse of this has led to the evolution of antimicrobial resistance. Antimicrobial peptides (AMPs) are natural defense elements present across many species including humans, insects, bacteria, and plants. Insect AMPs are our area of interest, because of their stronger abilities in host defense. We have deciphered AMPs from an endangered species *Parnassius bremeri*, commonly known as the red spotted apollo butterfly. It belongs to the second largest insect order Lepidoptera, comprised of butterflies and moths, and lives in the high altitudes of Russia, China, and Korea. We aimed at identifying the AMPs from the larvae stages. The rationale of choosing this stage is that the *P. bremeri* larvae development occurs at extremely low temperature conditions, which might serve as external stimuli for AMP production. RNA was isolated from larvae (L1 to L5) instar stages and subjected to next generation sequencing. The transcriptomes obtained were curated in in-silico pipelines. The peptides obtained were screened for requisite AMP physicochemical properties and in vitro antimicrobial activity. With the sequential screening and validation, we obtained fifteen candidate AMPs. One peptide TPS–032 showed promising antimicrobial activity against *Porphyromonas gingivalis*, a primary causative organism of periodontitis.

## 1. Introduction

Humans are privileged by having a strong innate and adaptive immune architecture, while smaller organisms like insects are solely dependent on innate immune systems. Defensive antimicrobial peptides (AMPs) are one of the key components of innate immune systems observed in different organisms. AMPs were proven effective against a broad range of pathogenic bacteria, fungi, parasites, and viruses [1]. Albeit their exceptional potency against pathogens, these have been little utilized until recently. The overuse or inappropriate dosage of classical antibiotics has developed a serious problem of anti-microbial resistance [2,3]. Thus, AMPs can be considered as an alternative pharmacological resource from nature confronting antibiotic resistant microorganisms.

Although AMPs are widely observed in both invertebrates and vertebrates, we are interested in studying insect AMPs as they are crucial for their survival in pathogen rich habitats [4,5]. *Parnassius bremeri* (*P*. *bremeri*), commonly referred as the red spotted apollo butterfly, has been chosen for this study. *P*. *bremeri* is listed as an endangered species, as their existence around the world has been significantly reducing because of global warming and shrinking of its habitat [6]. It belongs to the second largest insect order, Lepidoptera, which includes butterfly and moth species. Different families of AMPs (like cecropins, attacins, moricins, lebocins, gloverins) which are documented from this order, have been well summarized and discussed elsewhere [7,8,9]. Knowing the divergence and efficacy of Lepidopteran derived AMPs, we utilized the transcriptome of *P*. *bremeri* to identify novel AMPs. The identified peptides were then tested against diverse pathogenic bacterial and fungal species. 

Oral mucosa is a niche to diverse microorganism, and any alteration in the microbiome leads to diseases like dental caries, periodontitis, and gingivitis [10]. Periodontitis is an oral inflammatory disease caused by gram-negative bacteria, especially *Porphyromonas gingivalis*. The treatment for periodontitis involves mechanical therapy such as scaling and then antibiotics. However, improper use of antibiotics has caused the emergence of antimicrobial resistant strains. Thus, AMP can serve as an alternative form of treatment [11,12,13]. In this study we found that Peptide TPS–032 showed antimicrobial activity against *P. gingivalis*. Also, the peptides were tested for cytotoxicity using human keratinocyte (HaCaT) cells. HaCaT cell lines are highly proliferating epidermal cells with an almost normal phenotype. This cell line provides a virtually infinite supply of resembling cells, guaranteeing tremendous reproducibility, and is of great relevance to human epidermal-induced irritation. 

## 2. Materials and Methods

### 2.1. Strains Preparation

The bacterial and fungal strains used in this study were obtained from the KTCC (Korean Collection for Type Cultures), KACC (Korean Agricultural Culture Collection) or CCARM (Canadian Centre for Agri-Food Research in Health and Medicine). The bacterial or fungal strains were inoculated into the appropriate medium and grown at the temperatures shown in Table 1. All strains were stored in 20% glycerol at −70 °C.

### 2.2. P. bremeri Rearing and RNA Isolation

The *P. bremeri* rearing was followed, as reported earlier [6], in field conditions (Hweongsung, Korea). Briefly, eggs were manually attached to the fallen oak tree leaves in double net cages (0.1 × 0.3 mm mesh) for 180 days. The eggs were then transferred to young larval cage (40 × 50 × 70 cm), and the resulting newly hatching larvae were collected into a plastic petri dish (10 cm diameter × 4 cm height) with a supplement of the host plant, *Sedum kamtschaticum*. At the 4th instar (L4), the larvae were separated into 30 individuals and kept in a metal cage (71 × 51 × 88 cm covered with 1 × 1 mm metal mesh) with host plants. Total RNA was isolated from 1st instar to L1 to 5th instar to L5 stages using Trizol reagent (Invitrogen, Carlsbad, CA, USA), according to the manufacturer’s instruction. 

### 2.3. Next Generation Sequencing and Assembly

The integrity of isolated RNAs were analyzed using the Bioanalyzer 2100s system (Agilent technologies, Inc., Santa Clara, CA, USA). The RNA integrity Number (RIN) >7 was set as RNA QC to proceed to the next step of cDNA library construction (Truseq Stranded mRNA Prep Kit (Illumina Technologies, San Diego, CA, USA)). Further, constructed cDNA libraries were sequenced using an Illumina NovaSeq 6000 platform to generate 100 bp paired end reads. This was followed by quality checking by FASTQC [14], adaptor trimming using Trimmomatic [15] and de novo assembly in the Trinity program [16] with default parameters. A Cluster Database at High Identity with Tolerance (CD-HIT-EST) tool [17] was used to remove the redundancy transcripts with an identify threshold of 95% sequence similarity. The completeness of the assembled transcriptome was analyzed with gVolante against Arthropoda [18].

### 2.4. AMP Screening

The longest ORF per transcript was identified by the TransDecoder [16] to predict peptide and protein fragment. The AMP prediction was carried out as established previously. Briefly, the physiochemical properties (length, pI, charge), aggregation propensity (in vivo), aggregation propensity (in vitro), and antimicrobial region were determined by Pepstats [19], Tango [20], Aggrescan [21] and AMPA tools [22] respectively, with given cutoffs detailed in Table 2. The peptides which satisfied all of these conditions were considered for successive filtration. To find the novelty of the obtained peptides, the amino acid sequences were BLAST against CAMP (Collection of Anti-Microbial Peptides) [23], ADAM (A Database of Anti-Microbial Peptides) [24], and APD (The Antimicrobial Peptide Database) [25] databases. Peptides with a similarity score < 80 were considered novel. Finally, we used several classifiers from CAMP and ADAM databases, as mentioned in Table 2, to predict the given input peptide as AMP or non-AMP. The peptides which passed through all of these filters were then subjected to in house in-silico prediction strategies.

### 2.5. Peptide Synthesis and Antimicrobial Activity

Fifteen putative novel peptides were synthesized using solid-phase peptide synthesis methods at Lugen Sci Co. Ltd. (Bucheon, Korea). Then, each peptide was purified to >95% by high-performance liquid chromatography, and the purity was confirmed by mass spectrometry analysis. The peptides were dissolved in distilled water at a concentration of 1 mg/mL. To assess the anti-microbial activity, a broth microdilution method assay was used [26]. The bacterial or fungal strains were inoculated into the appropriate medium overnight at the proper culture temperature as shown in Table 1. The bacterial or fungal strains were adjusted at OD600 to 0.1 or 0.01, with the final compound concentrations ranging from 3.125 to 100 μg/mL. The growth control without treatment and the sterilized medium control were included in each experiment, and oxytetracycline or miconazole was used as the positive control. The growth rate was measured at OD600 using a microplate reader (BioTek Instruments, Seoul, Korea) at 24 h.

### 2.6. Cell Viability Assays

The cytotoxicity of the three peptides against HaCaT cells was tested using an MTT assay [27]. Briefly, HaCaT cells in Dulbecco’s Modified Eagle’s Medium (DMEM), at a density of 10^4^ cells per well of a 96-well plate, are cultured for 24 h. A serum-free medium containing various concentrations of peptides were added to the wells. After additional incubation for 24 h, MTT (3-(4,5-dimethyl-thiazol-2-yl)-2,5-diphenyltetrazolium bromide, Sigma) in PBS was added to a final concentration of 0.5 mg/mL, followed by incubation for 3 h at 37 °C. This solution was then removed, and cells were suspended in 100 mL of DMSO (Dimethyl Sulfoxide, Junsei) for 10 min. Absorbance was calculated from optical density (OD540) values measured using a microplate reader (BioTek Instruments, Seoul, Korea). Three independent experiments were carried out in triplicate.

## 3. Results

Three biological replicates for each larva (L1-L5) instar stage were sequenced. Due to lack of a reference genome, we tried de novo assembly of the *P. bremeri* transcriptome using a Trinity assembler. The assembly resulted in 48,672 unigenes with the average length of 1,710 base pairs. Unigenes with the length > 300 base pairs were considered for further processing. The completeness assessment score, or the BUSCO validation, was about 98.50%.

### 3.1. In Silico AMP Prediction

We have used next generation sequencing technology as a tool to decipher potent AMPs from red spotted apollo butterflies. The application of our in-silico AMP screening platform in insects has been successfully employed in our previous studies. Although AMPs are prevalently produced across species, certain structural features are important for their effective antimicrobial activity. Essentially the cationic and amphipathic structure is crucial for the bactericidal or fungicidal functions. These crucial physiological characteristics has been included in our screening schema that includes charge, isoelectric point, and antimicrobial spots (Table 1). Further, the naturally occurring AMPs mostly consist of 10 to 100 amino acid lengths [28]; we here considered the range of ≥2 to 50 amino acids to elude the high peptide manufacturing cost. We also measured the aggregation propensity in two different tools: Tango, which measures the aggregation in solution, and AGGRESCAN, to predict in vivo aggregation (bacterial cell membrane). Peptides which satisfy the given physicochemical propensity were then blasted against three antimicrobial databases to identify the novel peptide. Then, peptides were again run against supervised machine learning classifiers in CAMP and ADAM, such as Support Vector Machine (SVM), Random Forests (RF) and Artificial Neural Network (ANN) for AMP and non-AMP prediction. Peptides which satisfy all of the parameters were chosen for further downstream processing.

### 3.2. Antimicrobial Activity

The final peptides (Table 2) were again filtered with our in-house AMP prediction system (Korea patent application number: 10-2019-0019906). Fifteen peptides were finally selected and tested for anti-microbial activity against sixteen bacterial and eight fungal species. Out of the fifteen peptides, only three showed (TPS-029, TPS-032, TPS-035) positive antibacterial or antifungal effects (Table S1) in the screening test. Distinctively, the antimicrobial activity against *P. gingivalis* or *F. neoformans var. bacillispora,* or *P. guilliermondii* were noted. The broth microdilution method was used to identify the minimum inhibitory concentration of each peptide, presented here in Table 3. Oxytetracycline and miconazole were used as positive controls. Among the three peptides, TPS-032 were more effective against *P. gingivalis*. TPS-029 showed antifungal activity against *F. neoformans var. bacillispora*. The antifungal activities of TPS-032 and TPS-035 are displayed against *P. guilliermondii* (Table 3).

### 3.3. Cell Viability

**Figure 1:**
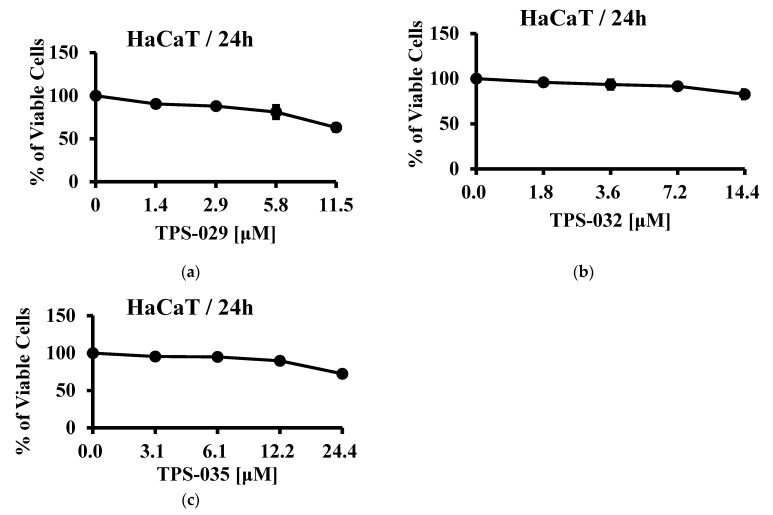
Cell viability assay. Effect of peptides (**a**) TPS-029 (**b**) TPS-032 and (**c**) TPS-035 on the growth of HaCaT cells at 24 h.

Although AMPs exhibit antibacterial or antifungal effects, cytotoxicity towards normal mammalian cells prevents its use as a pharmacological agent. Here, a human keratinocyte cell line (HaCaT) was used to test the cytotoxicity of the three peptides. TPS-035 showed cell viability above 80% at 14.4 μM, at which MIC against *P. gingivalis* was observed (Figure 1a). However, TPS-029 and TPS-032 at increased concentrations displayed reduced viable cells (Figure 1b,c).

## 4. Discussion

The quest for new AMP candidates is necessary to prevent or treat the evolving antimicrobial resistance. Pertaining to this, we have developed an in-silico AMP identification system harnessing genomic source, using previously identified potential AMP candidates from insects [4,5]. Insect AMPs are diversified in their structure, and show a broad range of activities such as being antibacterial, antifungal, antiviral, anticancer, and anti-parasitic. We report for the first time AMPs from red spotted apollo butterflies, an endangered species as listed in the International Union for Conservation of Nature and Natural Resource (IUCN) red list [6]. We have documented and deciphered their transcriptome in the process of identifying AMPs which might serve as a resource for future exploration. *P. bremeri* larva developmental stages occur during December to May in high altitudes, wherein extreme cold conditions prevail [6]. Thus, we are curious to identify the AMPs possibly involved in protecting the larvae.

The in-silico AMP identification schema includes the physicochemical propensity necessary for the AMP activity. Predominant characteristics of AMPs include cationicity, low aggregation in solution, higher aggregation in vivo, hydrophobicity, and amphipathicity [29,30,31]; all of these were considered in our screening platform. Although we attained relatively more candidates in the initial steps involving length, pI, charge, AMP spots, aggregation properties and trained classifiers from prominent AMP databases, the consecutive screening (Korea patent number: 10-2019-0019906) reduced the numbers considerably. Thus, stringent screening resulted in a few candidates to be tested in vitro. Fifteen peptides were tested against several bacterial and fungal strains, but only three peptides showed antibacterial and antifungal activity. *P. gingivalis* is a human pathogenic bacteria causing periodontitis [32,33], and its noted association with other disease like Alzheimer’s disease [34,35] is a serious threat to human health. Identifying novel drug candidates against *P. gingivalis* will add value to a treatment regimen. TPS-032 exhibited strong anti *P. gingivalis* activity. While all three peptides showed antifungal activity, they are less effective than the positive control. HaCaT cells exert normal epidermal phenotypes, and have a greater human relevance than animal derived cells. Hence, cytotoxicity towards HaCaT cells was measured for three peptide candidates. Little or no cytotoxicity was observed when treated with TPS-032, which shows its specificity towards *P. gingivalis*. In total, TPS-032 showed antibacterial and antifungal activity with less cytotoxicity. With further experimental validations, TPS-032 could serve as a potential lead candidate alone or in combination to treat *P. gingivalis* infections.

## Figures and Tables

**Table 1:** Strains used in this study and culture conditions.

Stains	Description	Medium	Temperature (°C)	Atmosphere
*Staphylococcus epidermidis*	KACC 12454	NB	30	Aerobic
*Klebsiella oxytoca*	KCTC 1686	NB	37	Aerobic
*Salmonella typhimurium*	CCARM 0240	NB	37	Aerobic
*Escherichia coli*	KACC 11598	TSB	37	Aerobic
*Enterococcus faecalis*	CCARM 5511	TSB	37	Aerobic
*Enterococcus faecium*	KACC 11954	TSB	37	Aerobic
*Pseudomonas aeruginosa*	KACC 14021	TSB	37	Aerobic
*Staphylococcus aureus*	CCARM 3505	TSB	37	Aerobic
*Streptococcus mutans*	KACC 16833	TSB	37	Aerobic
*Staphylococcus epidermidis*	KACC 13234	TSB	37	Aerobic
*Fusobacterium nucleatum* subsp. Nucleatum	KCTC 2640	BHI	37	Aerobic
*Actinomyces viscosus*	KCTC 9146	BHI	37	Anaerobic
*Propionibacterium acnes*	CCARM 9009	BHI	37	Anaerobic
*Porphyromonas gingivalis*	KCTC 5352	Modified TSB	37	Anaerobic
*Streptococcus sobrinus*	KCTC 5809	Modified TSB	37	Anaerobic
*Candida albicans*	KCTC 7965	YPD	30	Aerobic
*Candida tropicalis*	KCTC 7212	YPD	30	Aerobic
*Candida parapsilosis*	KACC 49573	YPD	30	Aerobic
*Candida tropicalis* var. tropicalis	KCTC 17762	YPD	30	Aerobic
*Candida parapsilosis* var. parapsilosis	KACC 45480	YPD	30	Aerobic
*Candida glabrata*	KCTC 7219	YPD	30	Aerobic
*Pichia guilliermondii*	KCTC 7211	YPD	30	Aerobic
*Filobasidiella neoformans* var. bacillispora	KCTC 17528	YPD	30	Aerobic

NB (Nutrient broth), TSB (Tryptic Soy Broth), BHI (Brain–Heart Infusion), Modified TSB (TSB supplemented with 2% sheep blood) and YPD (Yeast Peptone Dextrose).

**Table 2:** Antimicrobial peptide properties prediction and filtration.

Propensity	Tools	Descriptions/Parameters	Cutoff	No. of Sequences
		Total Protein fragments		266,300
Physicochemical	Pepstats	Peptide Length	≥2 to 50	189,818
	Pepstats	Charge	>0 (+)	128,625
	Pepstats	Isoelectric Point(pI)	≥8 to ≤12	88,951
	AMPA	Stretch	≥1	152,758
Aggregation (Invivo)	Tango	AGG	≤500	157,940
	Tango	Helix	≥0 Helix ≤25	179,511
	Tango	Beta	≥25 Beta ≤100	88,470
Aggregation (Invitro)	Aggrescan	Na4vSS	≥−40 Na4vSS ≤60	149,072
Similarity	BlastP	Similarity	<80	25,246
AMP	CAMP ADAM	Support Vector Machine (SVM) classifier	>0.5, AMP	7574
		Random Forest Classifier	>0.5, AMP	8571
		Artificial Neural Network (ANN) classifier	AMP	12,051
		Discriminant Analysis classifier	>0.5, AMP	9127
		Support Vector Machine (SVM) classifier	>0.5, AMP	17,976
Final				3570

CAMP—Collection of Anti-Microbial Peptides, ADAM—A Database of Anti-Microbial peptides.

**Table 3:** Minimum inhibitory concentrations. [μM (μg/mL)].

	Sequence	Mw (Da)	*Porphyromonas gingivalis* (KCTC5352)	*Filobasidiella neoformans* var. Bacillispora (KCTC17528)	*Pichia guilliemondii* (KCTC7211)
Oxytetracycline		460.4	108.6 (50)	ND	ND
Miconazole		416.1	ND	7.5 (3.125)	7.5 (3.125)
TPS-029	RLFNYGLFSSKIIKHTIK	2165.76	>46.2 (>100)	23.1 (50)	>46.2 (>100)
TPS-032	RVLTHVFKCKLKLR	1741.41	14.4 (25)	>57.4 (>100)	28.7 (50)
TPS-035	RCCKLVFR	1024.42	>97.6 (>100)	>97.6 (>100)	97.6 (100)

ND—not detected, Mw—molecular weight.

## Data Availability

The transcriptome data is deposited in the National Center for Biotechnology (NCBI) Sequence Read Archive (SRA) under the accession number SRA1102671.

## Supplementary Materials

The following are available online at https://www.mdpi.com/article/10.3390/insects12050466/s1, Table S1: peptide screening for antibacterial and antifungal activity.
